# Preparation and Application of Light-Colored Lignin Nanoparticles for Broad-Spectrum Sunscreens

**DOI:** 10.3390/polym12030699

**Published:** 2020-03-21

**Authors:** Sang Cheon Lee, Eunjin Yoo, Sang Hyun Lee, Keehoon Won

**Affiliations:** 1 Department of Chemical and Biochemical Engineering, Dongguk University-Seoul, 30 Pildong-ro 1-gil, Jung-gu, Seoul 04620, Korea; lsc0946@gmail.com; 2 Department of Biological Engineering, Konkuk University, 120 Neungdong-ro, Gwangjin-gu, Seoul 05029, Korea; ejyoo0805@naver.com (E.Y.); sanghlee@konkuk.ac.kr (S.H.L.)

**Keywords:** lignin, color, nanoparticles, cellulolytic enzyme, sunscreens

## Abstract

Recently, natural sun blockers have been drawing considerable attention because synthetic UV filters could have adverse effects not only on humans but also on the environment. Even though lignin, the second most abundant renewable resource on earth, is a natural UV-absorbing polymer, its unfavorable dark color hampers its applications in sunscreens. In this work, we obtained light-colored lignin (CEL) from rice husks through cellulolytic enzyme treatment and subsequent solvent extraction under mild conditions and compared CEL to technical lignin from rice husks using the International Commission on Illumination L*a*b* (CIELAB) color space. Spherical nanoparticles of CEL (CEL-NP) were also prepared using a solvent shifting method and evaluated for broad-spectrum sunscreens. A moisturizing cream blended with CEL-NP exhibited higher sun protection factor (SPF) and UVA PF (protection factor) values than that with CEL. In addition, CEL-NP had synergistic effects when blended with an organic UV-filter sunscreen: CEL-NP enhanced the SPF and UVA PF values of the sunscreen greatly. However, there was no synergistic effect between CEL-NP and inorganic sunscreens. We expect nanoparticles of light-colored lignin to find high-value-added applications as a natural UV-blocking additive in sunscreens and cosmetics.

## 1. Introduction

Since long-term exposure to the sun’s UV radiation can result in skin aging, sunburn, and skin cancer, interest in sunscreens has been increasing. Active ingredients of sunscreens are divided into two categories: organic and inorganic. Organic UV filters (e.g., oxybenzone and octinoxate) absorb UV rays, whereas inorganic UV filters (e.g., TiO_2_ and ZnO nanoparticles) not only absorb but also scatter them [1]. These conventional sunscreen ingredients based on synthetic compounds have been reported to have adverse impacts not only on humans but also on the environment [2]. It is a recent trend to use natural ingredients in the cosmetic market. Therefore, many cosmetic companies have been making an effort to replace synthetic sunscreening agents with natural and economical substances.

Lignocellulosic biomass is the most abundant and highly renewable natural resource on earth and primarily consists of cellulose, hemicellulose, and lignin. Lignin is a heterogeneous phenolic biopolymer formed by radical co-polymerization of three different phenylpropane monomers: *p*-coumaryl alcohol, coniferyl alcohol, and sinapyl alcohol [3]. Due to the UV-absorbing functional groups of lignin (phenolic, ketone, etc.), lignin has been used for various composite films with UV barrier properties [4,5]. In addition, lignin was successfully introduced as a natural sun blocker for broad-spectrum sunscreens [6]. However, its undesirable dark color is the biggest obstacle to the market promotion of lignin-based sunscreens [7,8]. Native lignin in lignocellulosic biomass undergoes structural changes and turns dark in color during separation processes, which are generally operated under harsh conditions such as high temperature and extreme pH values [9,10,11,12]. Recently, we extracted lignin under mild conditions (at room temperature with neutral solvents) and applied the obtained light-colored lignin to sunscreens [13]. Additionally, without isolating lignin from biomass, we introduced lignin-containing wood powders themselves as a natural sunscreen ingredient. The wood powder particles were not bigger than microbeads widely used in cosmetic products [14].

In this work, we prepared nanoparticles of light-colored lignin in order to improve its UV blocking ability and apply it to sunscreens. The light-colored lignin was first obtained from rice husks in a high yield using a cellulolytic enzyme, then quantitatively compared in color to technical lignin from rice husks. The lignin nanoparticles were examined in terms of size, shape, and UV transmittance in cosmetic creams. The effects of their addition to commercial sunscreens on UV protection performance were also investigated.

## 2. Materials and Methods

### 2.1. Materials

Conventional organosolv lignin (OL) extracted from rice husks (RH) was provided by SugarEn (Yongin, Korea). Tetrahydrofuran (THF) was from Avantor (Radnor, PA, USA) and used without further purification. The pure cream used was NIVEA refreshingly soft moisturizing cream. Organic and inorganic UV filter sunscreens were BIOTHERM Lait Solaire Hydratant (SPF 15), and ATTITUDE 100% Mineral (SPF 15), respectively. Their active and inactive ingredients can be found in the Supplementary Materials.

### 2.2. Preparation of Light-Colored Lignin

Light-colored lignin was prepared from rice husks following the previous procedure [15,16] with slight modifications. Rice husks were shredded with a home blender (ProBlend 6, Philips, Amsterdam, Netherlands) and then sieved to obtain a powder of less than 300 μm. The impurities of rice husk powders were removed by washing with 95% ethanol. Dried rice husk powders were milled with a planetary ball mill (Pulverisette 6, FRITSCH, Idar-Oberstein, Germany) at 500 rpm for 24 h. Milled powders of rice husk (3 wt %) were enzymatically hydrolyzed using 87 μL cellulase (Novozym 50199, Novozymes, Copenhagen, Denmark) per gram of rice husk powder in a 0.1 M citrate phosphate buffer (pH 5.0) at 37 °C for 24 h. Enzymatically hydrolyzed rice husk powder was washed by centrifugation and then freeze-dried. One gram of freeze-dried powder was mixed with 10 mL of 95% dioxane and stirred at room temperature for 24 h to extract lignin. The dioxane mixture containing lignin was separated by centrifugation and then evaporated. Extracted lignin was further purified by dissolution in 90% acetic acid followed by precipitation in water. Precipitated lignin, which is called cellulolytic enzyme lignin (CEL), was freeze-dried for further use.

### 2.3. Color Analysis and FTIR Spectroscopy of Light-Colored Lignin 

Color evaluation of lignin was conducted using the CM-2600d Spectrophotometer (Konica Minolta, Tokyo, Japan) with two different modes: specular component included (SCI) and specular component excluded (SCE). SCI is used to evaluate the actual color of an object including specular and diffuse reflected light, while SCE is used to determine the appearance of an object’s color by excluding any specular reflected light. In the International Commission on Illumination L*a*b* (CIELAB) color space, the L* value represents a bright behavior of a sample: white when L* = 100 and black when L* = 0; +a* is a red direction and −a* is a green direction; +b* is a yellow direction and −b* is a blue direction. A total color difference value (Δ*E*) is defined as follows:

Δ*E* = [(ΔL*)^2^ + (Δa*)^2^ + (Δb*)^2^]^1/2^ (1)

where ΔL*, Δa*, and Δb* are the differences in L*a*b* values between a reference and a sample. The color parameters were measured at 4 points for each sample and averaged. Fourier-transform infrared (FTIR) spectroscopy was also carried out using the TENSOR 27 FTIR Spectrometer (Bruker, Billerica, MA, USA). The FTIR spectra were recorded in the range of 4000−400 cm^−1^ using an attenuated total reflection (ATR) method.

### 2.4. Preparation of Lignin Nanoparticles

Cellulolytic enzyme lignin nanoparticles (CEL-NP) were fabricated using a solvent shifting method combined with ultrasonication [17]. CEL (50 mg) was completely dissolved in 50 mL of THF-water mixture (7:3 *v/v*). To the CEL solution (1 mg/mL), magnetically stirred at 800 rpm, was added 150 mL of deionized water at a dilution rate of 20 mL/s. The suspension was mixed for 1 h and then subjected to ultrasonication for 20 min (150 W, 20 kHz). The suspension was centrifuged at 13,000 g for 30 min, and the precipitate was washed with deionized water to collect CEL-NP, which were then dried in a vacuum desiccator for 24 h. The yield of nanoparticles was calculated from weight of nanoparticles after drying.

### 2.5. Characterization of CEL-NP

Before centrifugation, aliquots of CEL-NP suspension were used as samples. The CEL-NP size was analyzed using a particle size analyzer (Scatter Scope1, K1 Solution, Seoul, Korea). Scanning electron microscope (SEM) images were observed using the JSM-7800F (JEOL Ltd., Tokyo, Japan). Samples were deposited by dropping the CEL-NP suspension on a clean silicon wafer surface (0.25 mm^2^). After drying at 40 °C, the surface was coated with platinum for 50 s. SEM images were scanned at an acceleration voltage of 10 kV using a LED detector. 

### 2.6. Compositional Analysis of Rice Husks and the Prepared Lignins

The chemical compositions of the rice husks and the prepared lignins were analyzed according to the National Renewable Energy Laboratory (NREL) method [18]. Briefly, the samples were treated with 72% (*v/v*) sulfuric acid at 30 °C for 2 h, followed by dilute acid (4%) at 121 °C for 1 h. The hydrolysis products (glucose, xylose, galactose, arabinose, and mannose) were determined using an HPLC (Young-Lin Model YL9100, Anyang, Korea) equipped with a refractive index (RI) detector and a Shodex SP0810 column operated at 85 °C. The mobile phase consisted of deionized water with a flow rate of 0.6 mL/min. The glucan content was calculated as the glucose content multiplied by the conversion factor 0.90. The amount of hemicellulose was calculated as the total amount of xylose, galactose, arabinose, and mannose multiplied by the conversion factor 0.88. The filtered solid residue after acid hydrolysis was dried at 105 °C and was used to ascertain the acid-insoluble lignin. The amount of ash was then determined by burning the solid residue at 575 °C in a muffle furnace. The acid-soluble lignin was measured using a UV–Vis spectrophotometer at 240 nm with an extinction coefficient value of 106 L·g^−1^·cm^−1^. The lignin purity was calculated using the following equation:

(2) $$\mathrm{Lignin}\;\mathrm{purity}\;\left(\%\right)=\frac{\mathrm{Weight}\;\mathrm{of}\;\mathrm{acid}\;\mathrm{soluble}\;\mathrm{lignin}+\mathrm{Weight}\;\mathrm{of}\;\mathrm{acid}\;\mathrm{insoluble}\;\mathrm{lignin}}{\mathrm{Weight}\;\mathrm{of}\;\mathrm{dried}\;\mathrm{sample}}\times 100$$

### 2.7. Preparation and UV Transmittance Measurement of CEL-NP-Blended Creams

The CEL-NP were blended with the pure cream and the sunscreens using a magnetic stirrer at 600 rpm for 24 h. The whole blending process proceeded at room temperature in a dark room. The CEL-NP-blended samples were applied at 2 mg/cm^2^ to the 3M Transpore Tape (7.5 cm^2^) that was attached to the surface of a quartz plate. They were then spread over the tape by slowly rubbing the slide surface with a thimble-coated finger. Then, the prepared samples were dried in a dark room to block light for 20 min. UV transmittance was measured using the Cary 50 spectrophotometer equipped with a solid sample holder (Agilent Technologies, Santa Clara, CA, USA). Four spots were measured for each sample, and transmittance data were scanned in the range from UVB (290–320) to UVA (320–400 nm). After measuring the UV transmittance, in vitro evaluation of sun protection factor (SPF) was conducted using the following equation [7,19,20]:

(3) $$\mathrm{SPF}\;=\overset{400}{\underset{290}{\sum }}E_{\lambda }S_{\lambda \;}/\overset{400}{\underset{290}{\sum }}E_{\lambda }S_{\lambda }\;T_{\lambda }$$

where *E_λ_* is erythemal spectral effectiveness, *S_λ_* is solar spectral effectiveness, *T_λ_* is spectral transmittance of sample.

UVA protection factor (UVA PF) was also calculated using the following equation [21]:

(4) $$\mathrm{UVA}\;\mathrm{PF}\;=\overset{400}{\underset{320}{\sum }}E_{\lambda }I_{\lambda }\Delta \lambda /\overset{400}{\underset{320}{\sum }}E_{\lambda }I_{\lambda }\;T_{\lambda }\Delta \lambda$$

where *I_λ_* is biological action spectrum for UVA. However, Ferrero et al. suggested that if ∆*λ* is small enough, the equation can be simplified. In that case, both *E_λ_* and *I_λ_* are equal to unity for all UVA wavelengths [21].

## 3. Results and Discussion

### 3.1. Preparation and Color Evaluation of Light-Colored Lignin

In our previous study, milled wood lignin (MWL), which was obtained through 1,4-dioxane extraction at room temperature after ball milling, was used as light-colored lignin. Even though MWL was bright, unlike conventional lignins, its yield was low (about 5% based on the biomass weight). In this work, lignin was extracted with the solvent at room temperature in a manner similar to MWL, but ball-milled powder was treated with cellulolytic enzyme in order to increase the yield. The yield of CEL can be higher than that of MWL because enzymatic cellulose hydrolysis allows more lignin to separate from biomass [22]. The yield of CEL extracted from RH in this work was found to be 9.5% based on the biomass weight.

The chemical composition of CEL was evaluated according to the NREL procedure [18], and for comparison, RH and OL isolated from RH at high temperature (typically 180 °C) were also analyzed in Table 1. The lignin content of RH was about 23%, close to the average of 22%, and the chemical composition of RH may depend on the growth conditions of rice [23]. CEL had a purity of about 85% and contained a higher level of associated carbohydrates than OL. It is well known that lignin is always associated with carbohydrates (particularly with hemicellulose) through covalent linkages. Since CEL was isolated under mild conditions, this lignin–carbohydrate complex may be preserved more than in OL [16].

**Figure 1:**
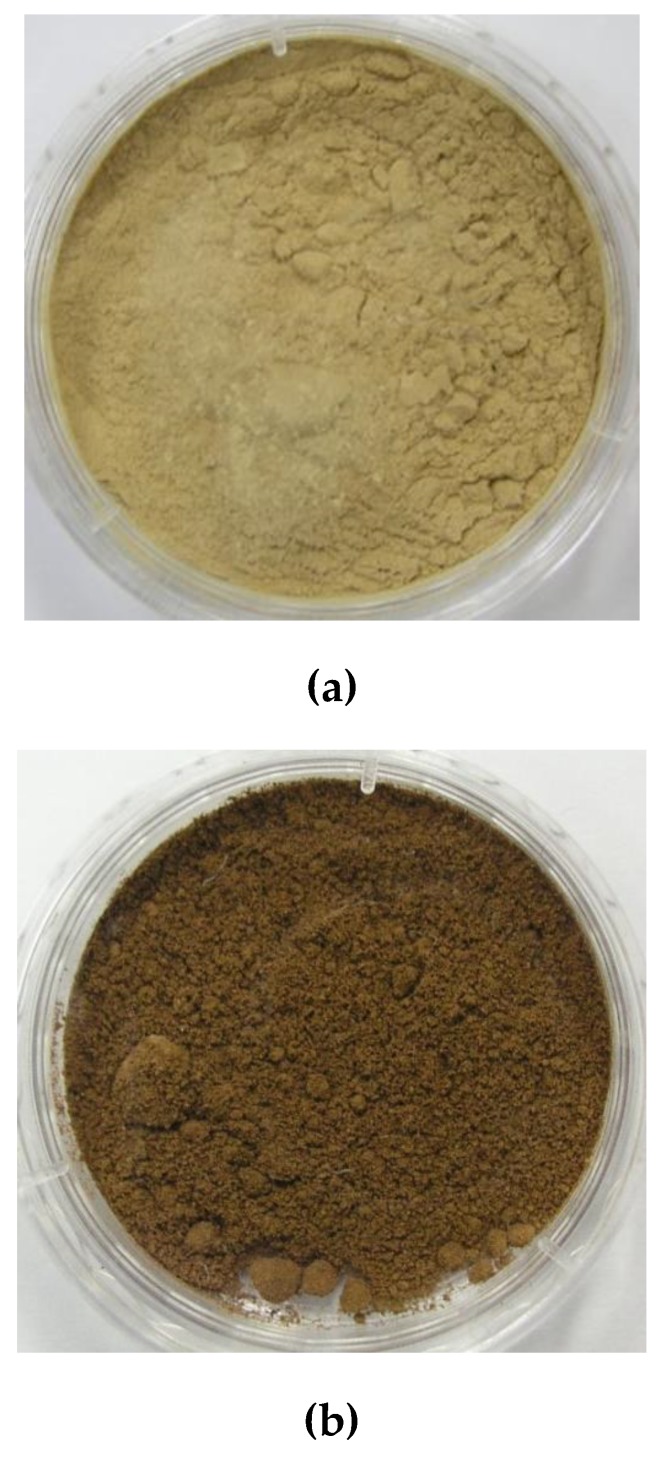

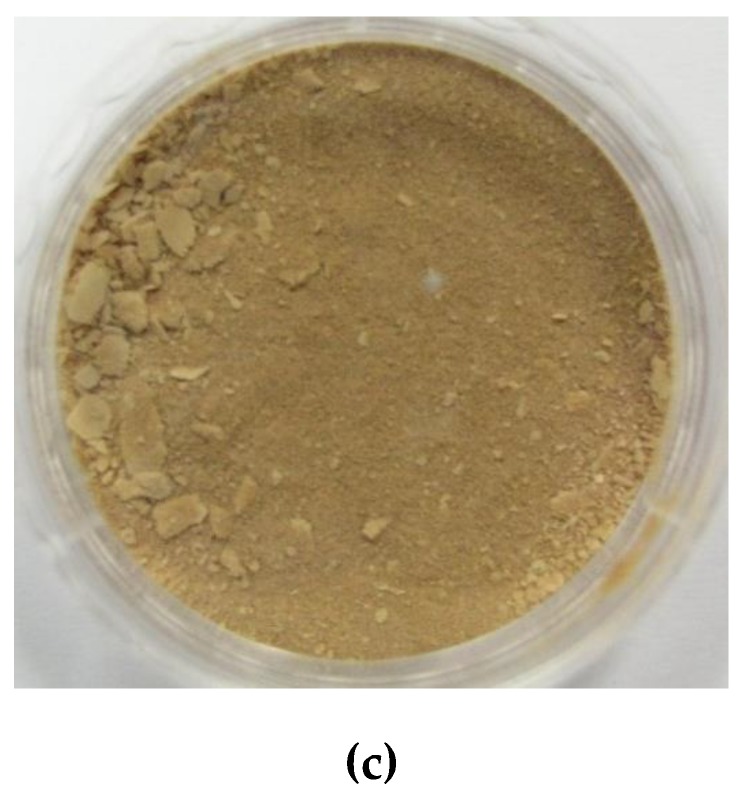
Photographs of (**a**) RH, (**b**) OL, and (**c**) CEL.

The color of CEL was observed with the naked eye and compared with that of RH and OL. Their photographs in Figure 1 show that CEL was as bright as RH and did not discolor significantly like MWL, whereas OL was dark, unlike RH, indicating lignin discolored during separation. Their colors were also evaluated quantitatively using the L*a*b* color space as shown in Table 2. The lightness (L*) and redness (a*) values of CEL were similar to those of RH. The L* value of CEL was much higher than that of OL, and the a* value of CEL was lower than that of OL. This is because CEL was prepared under far milder conditions than OL. It is generally accepted that wood is discolored by thermal degradation and/or oxidation of its components (lignin and hemicellulose). Lignin darkened and reddened after heat treatment, probably because of formation of condensation products and degradation and/or oxidation products, such as quinone-like substances [10]. The yellowing of bleached chemical pulp proceeded during heat treatment by decay of mainly hemicellulose, which is thermally less stable than cellulose [24]. Thermal decomposition of hemicellulose and cellulose occur with high intensity at 180 and 340 °C, respectively [25].

**Figure 2:**
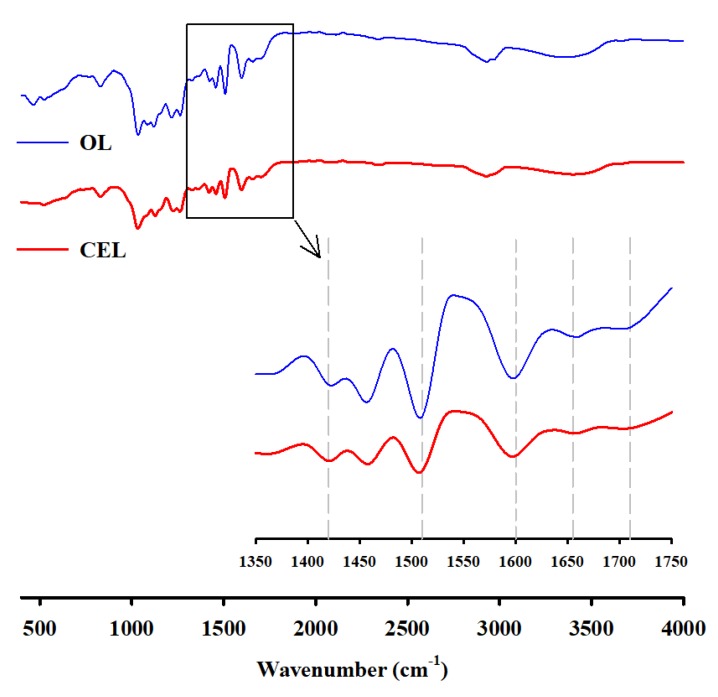
FTIR spectra of OL and CEL.

We measured the FTIR spectra in order to explain the color differences between CEL and OL, as shown in Figure 2. It was found that peak intensities of OL were higher than those of CEL mainly in the range of 1300 to 1800 cm^−1^. Absorption peaks of the aromatic skeleton vibration at 1510 and 1600 cm^−1^ can be intensified by cleavage of the abundant β–O–4 linkage and/or condensation reactions, which might result in the increase in phenolic OH groups (auxochromes) and/or the formation of conjugated structures such as coniferaldehyde (chromophores). The peak (1710 cm^−1^) assigned to C=O stretching in α, β-unsaturated aldehyde or unconjugated ketone or ester (unsaturated) may contribute to darkening. The intensified peak at 1650 cm^−1^ assigned to C=O stretching in conjugated *p*-substituted aryl ketones can be attributed to the formation of quinoid structures, which is responsible for the increased redness (a*) of OL. The peak at 1425 cm^−1^ is enhanced by the formation of new conjugated double bonds by heat treatment [10,26,27,28].

### 3.2. Preparation and Characterization of CEL-NP

There are several methods for fabrication of lignin nanoparticles (e.g., solvent shifting, pH shifting, spray freezing) [29,30,31,32]. CEL-NP were prepared by a solvent shifting method in which addition of antisolvent to lignin solution causes formation of spherical particles due to the minimization of surface energy [30]. CEL was completely dissolved in an aqueous THF mixture (30 vol % water) and then to this lignin solution was added water as antisolvent. The final water content in the lignin solution was 82.5 vol %. As the water content in the lignin solution increases gradually, the solvent quality becomes unfavorable for lignin solubilization and hence, lignin molecules begin to assemble due to hydrophobic interaction. When the water content reaches a critical value, lignin starts to form colloids in the solution. In the case of acetylated alkali lignin, the critical water content was 44 vol %, and the water content for the colloid completion was 67 vol % [33]. Lignin nanoparticles were prepared by the solvent shifting method in yields ranging from 33% to 91% depending on the production conditions and the lignin type [29]. The yield of CEL-NP prepared in this work was 45–47%. Compositional analysis of CEL-NP revealed that the purity of CEL-NP was about 90%, which was higher than that of CEL (about 85%) in Table 1. This is because the glucan and hemicellulose contents decreased, indicating that the carbohydrates were removed to some degree during the CEL-NP preparation.

**Figure 3:**
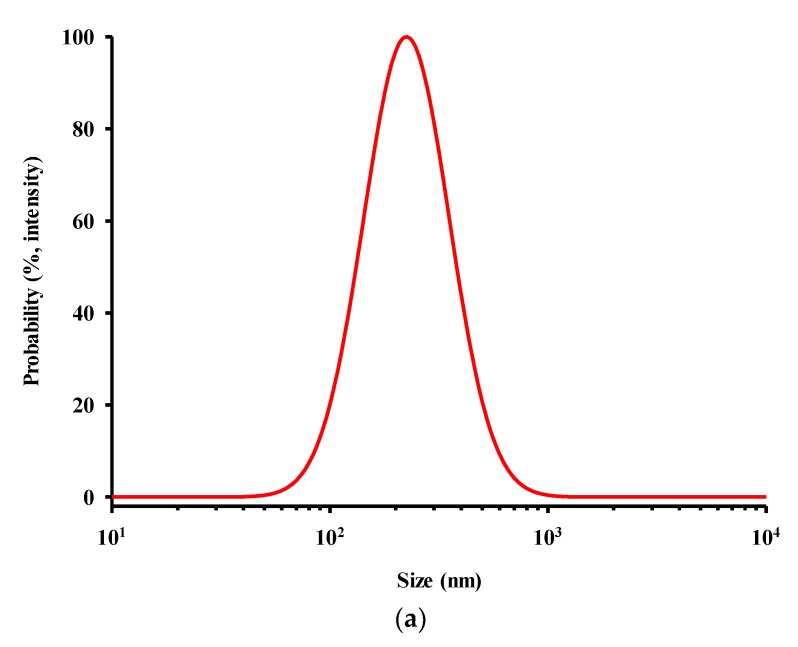

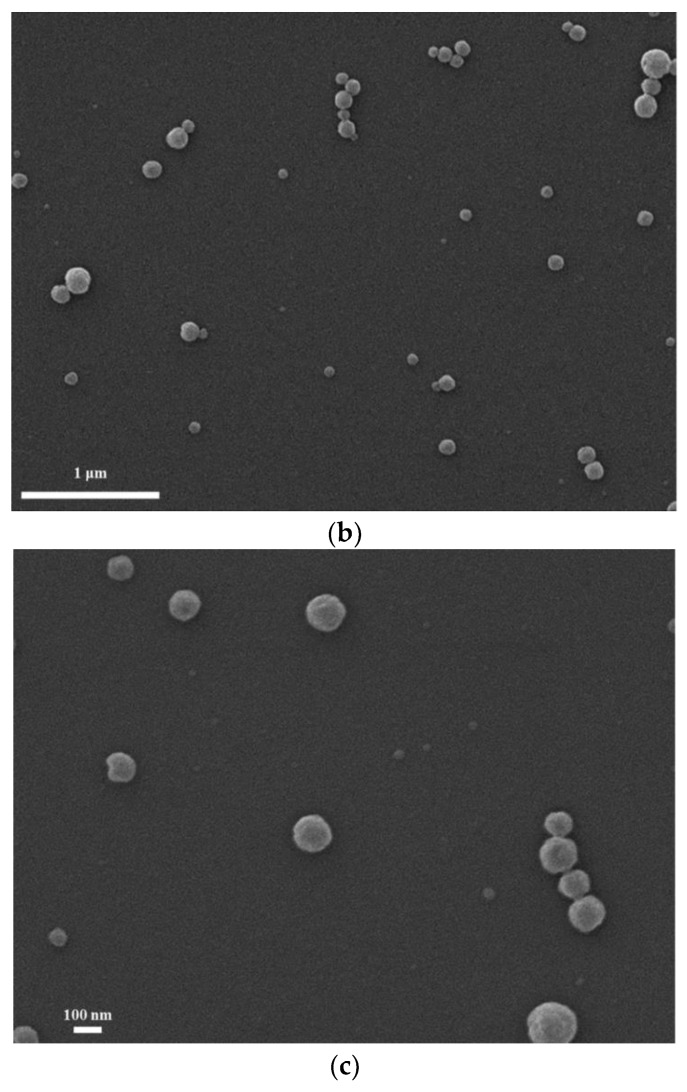
(**a**) Size distribution of CEL-NP(cellulolytic enzyme lignin nanoparticles); (**b**) SEM image of CEL-NP, magnification ×25,000; and (**c**) SEM image of CEL-NP, magnification ×50,000.

The size and shape of CEL-NP were examined using a particle size analyzer and SEM. Figure 3a indicates the average diameter of CEL-NP was 225 nm, and Figure 3b,c shows that CEL-NP were spherical solid particles. The size and shape of lignin nanoparticles are heavily dependent on their fabrication conditions. The size of lignin particles obtained by the solvent shifting method ranged from 41 to 2700 nm [29]. The particle size increased with increases in the initial lignin concentration and decreases in the dilution rate of the lignin solution [29,30]. Using lower initial lignin concentration and higher addition rate of antisolvent led to hollow nanoparticles, not solid nanoparticles [29,34].

### 3.3. UV Blocking Performance of CEL-NP-Blended Cream

**Figure 4:**
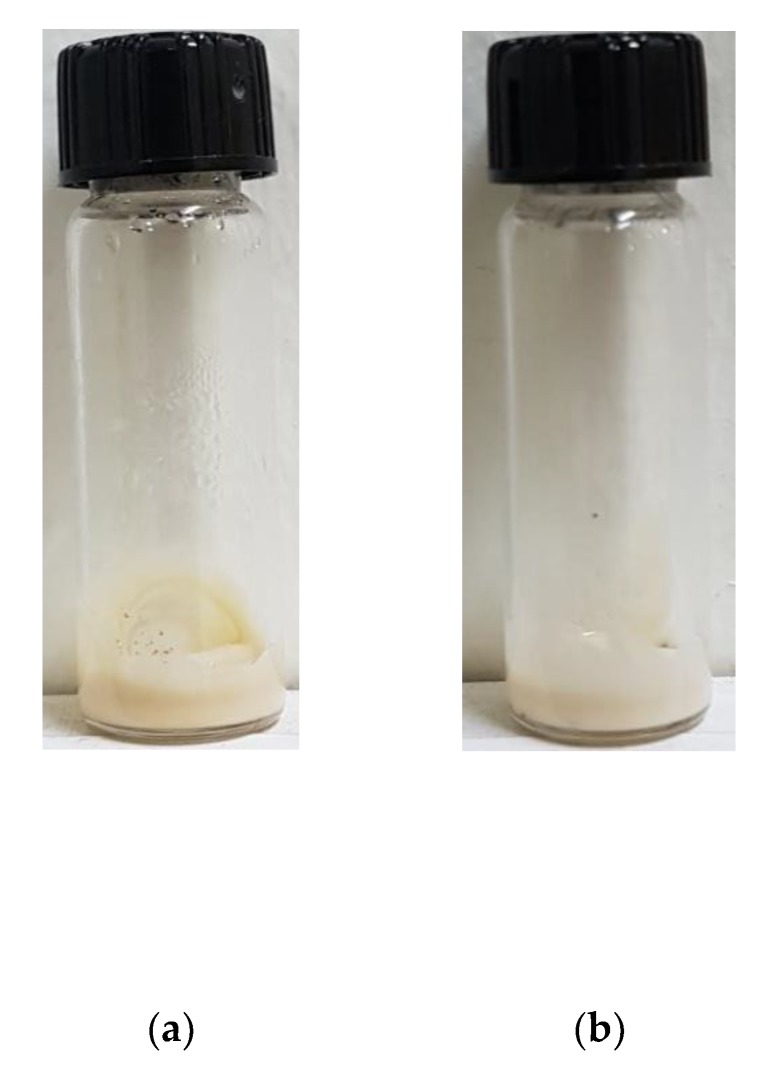

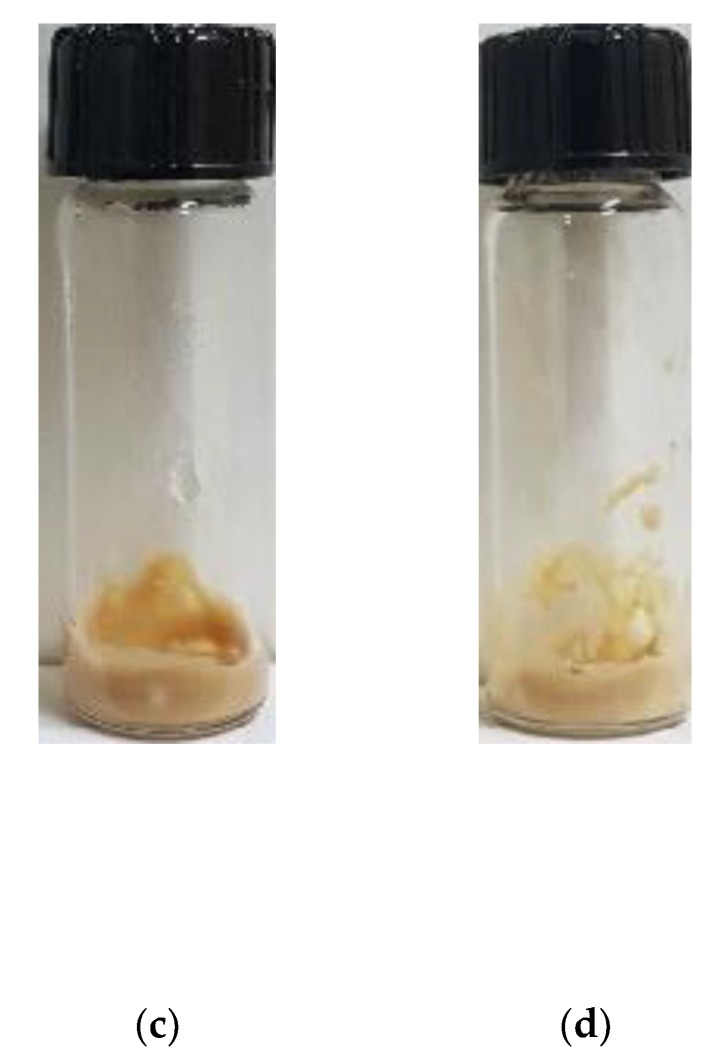
Appearance of the moisturizing cream blended with 1 wt % lignin: (**a**) CEL and (**b**) CEL-NP and with 5 wt % lignin: (**c**) CEL and (**d**) CEL-NP.

**Figure 5:**
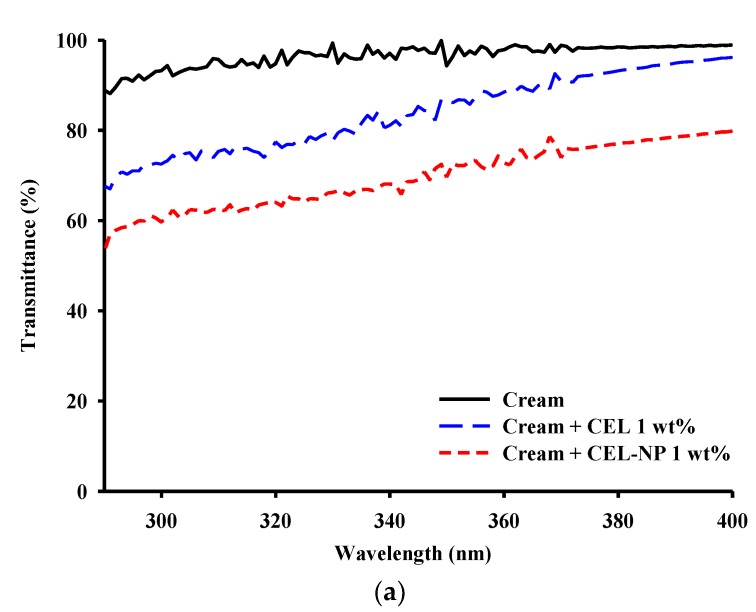

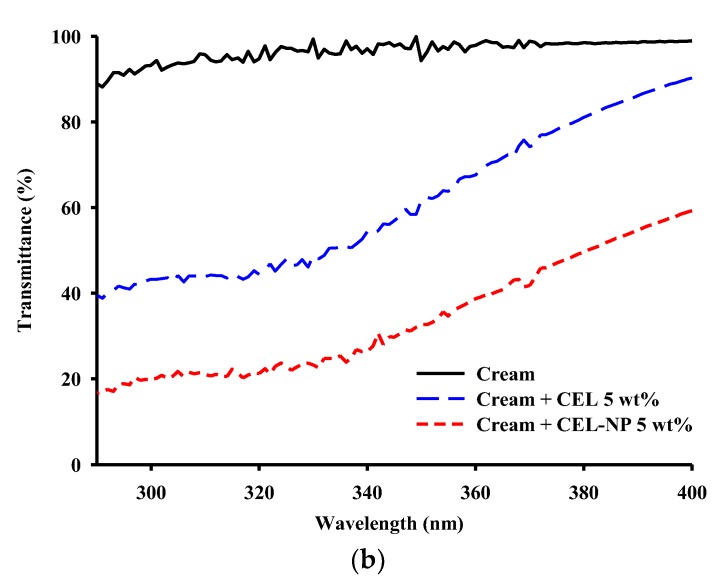
UV transmittances of the creams blended with (**a**) 1 and (**b**) 5 wt % CEL and CEL-NP.

In order to apply CEL and CEL-NP to sunscreens, they were blended with the commercial moisturizing cream (NIVEA) at concentrations of 1 and 5 wt %, and UV transmittances of the blended creams were measured. First, as far as color is concerned, CEL and CEL-NP-blended creams were not dark enough to be rejected by consumers as shown in Figure 4. Moreover, the CEL-NP creams in Figure 4b,d were lighter in color than the CEL creams in Figure 4a,c [35]. This may be because the apparent color of lignin is dependent not only on the structure but also on the bulk density on a macroscopic scale [36]. UV transmittance spectra of the pure cream and the lignin creams are presented in Figure 5. The native cream without lignin showed negligible absorbance in the UVA and UVB areas. However, the added lignins significantly decreased the transmittance of the cream across the range of UVA and UVB. In particular, the UV transmittance of the cream blended with CEL-NP became much lower. Table 3 shows the UV protection factor values of the cream blended with CEL and CEL-NP (1 and 5 wt %). SPF and UVA PF of the pure cream were 1.1 ± 0.0 and 1.0 ± 0.0, respectively, indicating no UV blocking. However, when the lignins were added to the cream, both the values increased in proportion to lignin amounts. As shown in Table 3, at 5 wt %, the CEL-NP cream exhibited SPF and UVA PF values about twice as high as those of the CEL cream. Similarly, the creams blended with enzymatic hydrolysis lignin and organosolv lignin nanoparticles also exhibited higher SPF values than those with lignin powders [35]. The better UV protection performance of the CEL-NP cream may be attributed to π–π interactions of lignin molecules in nanoparticles. Various noncovalent interactions are responsible for lignin aggregation in solvents: π–π interactions, electrostatic attractions, and van der Waals forces. It is generally known that electrostatic and van der Waals forces are not strong enough to form lignin nanoparticles. The strong π–π interactions among the abundant aromatic rings of lignin macromolecules are considered to be the main driving force for nanoparticle formation [17,33,37]. The π–π stacking of aromatic groups can form two distinct types of aggregates: J- and H-aggregates. They are determined by the tilt angle between the molecular axis connecting the chromophores and transition dipole moment of the chromophore. When the angle is less than 54.7°, the J-aggregate is formed. In contrast, when the angle is more than 54.7°, the H-aggregate is formed [38]. It was demonstrated that lignin formed the J-aggregate by the π–π interactions in aqueous organic solvents [39,40]. Unlike the H-aggregation, the J-aggregation decreases the energy required for π–π* transition, which may account for an increase in UV absorption [41].

### 3.4. Synergistic Effects of CEL-NP with Sunscreens

**Figure 6:**
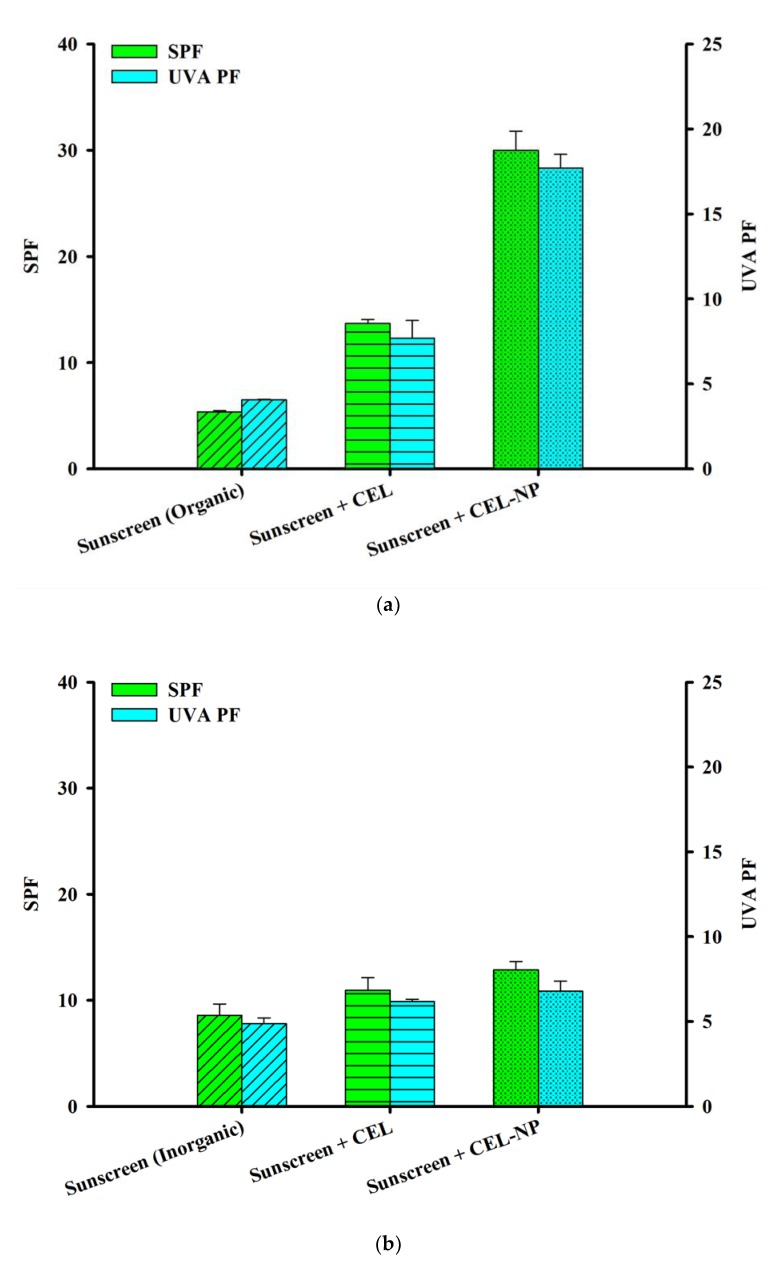
SPF and UVA PF values of lignin-blended sunscreens (5 wt %) based on (**a**) organic UV filters and (**b**) inorganic UV filters.

It was found that lignin could boost SPF of sunscreens considerably when it was added to conventional sunscreens [6,41]. In order to check if lignin nanoparticles also have this synergistic effect with sunscreens, CEL and CEL-NP were blended at 5 wt % with the BIOTHERM SPF 15 sunscreen, which contains a few aromatic compounds as active ingredients (octocrylene, ethylhexyl salicylate, butyl methoxydibenzoylmethane, and ethylhexyl triazone). In Figure 6a, SPF and UVA PF values of the lignin-blended sunscreens are shown. When CEL was added, the UV protection factor values approximately doubled due to their synergistic effect. In the case of CEL-NP, these values jumped about fivefold overall (from 5.4 to 30.0 for SPF; from 4.1 to 17.7 for UVA PF). Recalling SPF and UVA-PF values of the 5 wt % CEL-NP cream were 4.3 ± 0.4 and 2.6 ± 0.2, respectively, Figure 6a clearly demonstrates that CEL-NP has a synergistic effect with sunscreens, as CEL does. This synergy was explained by J-aggregation of the aromatic rings of lignin and active ingredients in sunscreens. As discussed earlier, the J-aggregation can increase UV absorbance [41].

In order to check this explanation, CEL and CEL-NP were blended with the ATTITUDE SPF 15 sunscreen, which contains not aromatic compounds but zinc oxide as active ingredients. Interestingly, addition of the lignin (5 wt %) slightly increased SPF and UVA PF values of the sunscreen (Figure 6b), which is in marked contrast to results shown in Figure 6a. This implies that both CEL and CEL-NP exhibit no synergistic effect with the mineral sunscreen, indicating that aromatic active components in sunscreens are responsible for the synergy. 

## 4. Conclusions

Spherical nanoparticles of light-colored lignin isolated under mild conditions (CEL-NP) were fabricated and applied as a natural sunscreening agent for the first time. A moisturizing cream blended with 5 wt % CEL-NP exhibited SPF and UVA PF values about twice as high as those of a cream blended with CEL. Furthermore, CEL-NP had synergistic effects with an organic UV-filter sunscreen: addition of 5 wt % CEL-NP increased the SPF and UVA PF values of the sunscreen about fivefold overall. There was no synergistic effect between CEL-NP and inorganic sunscreens. We expect light-colored lignin nanoparticles to find high-value-added applications as a natural UV-blocking additive in sunscreens and cosmetics.

## Figures and Tables

**Table 1:** Chemical composition of rice husks and prepared lignins.

Sample	Composition (%)			
	Glucan	Hemicellulose	Lignin	Ash
**RH**	31.7	15.8	23.3	20.0
**OL**	1.2	6.6	84.5	ND
**CEL**	2.2	8.9	85.1	ND
**CEL-NP**	1.0	6.3	89.5	ND

**Table 2:** Color coordinates and color differences of RH (rice husks), OL (organosolv lignin), and CEL (cellulolytic enzyme lignin).

Sample	SCI				SCE			
	L*	a*	b*	Δ*E*	L*	a*	b*	Δ*E*
**White reference ^a^**	99.5	−0.1	−0.1	0.0	97.3	−0.1	0.1	0.0
**RH**	71.6	4.6	21.3	35.5	65.3	5.2	26.5	41.8
**OL**	55.1	7.5	15.9	47.8	44.4	10.1	27.1	60.2
**CEL**	66.8	5.4	15.9	36.8	59.9	6.3	20.3	42.9

^a^ The plate for white calibration as a spectrophotometer accessory.

**Table 3:** SPF (sun protection factor) and UVA PF (protection factor) of the moisturizing cream * blended with different amounts of CEL and CEL-NP.

Sample (wt %)	CEL		CEL-NP	
	SPF (290–400 nm)	UVA PF (320–400 nm)	SPF (290–400 nm)	UVA PF (320–400 nm)
**1**	1.3 ± 0.1	1.3 ± 0.1	1.6 ± 0.1	1.4 ± 0.0
**5**	2.2 ± 0.1	1.5 ± 0.0	4.3 ± 0.4	2.6 ± 0.2

* SPF and UVA PF of the moisturizing cream were 1.1 and 1.0 ± 0.0, respectively.

## Supplementary Materials

The active and inactive ingredients of the moisturizing cream and the sunscreens are available online at https://www.mdpi.com/2073-4360/12/3/699/s1.
