# Supplementary material for: Preparation and Application of Light-Colored Lignin Nanoparticles for Broad-Spectrum Sunscreens
Source: Polymers (Basel). 2020 Mar 21;12(3):699. doi: 10.3390/polym12030699 (PMC7183278; doi:10.3390/polym12030699)
Supplement: Supplementary file 1 [file polymers-12-00699-s001.zip › polymers-744325-supplementary.docx]

**Supplementary Materials**

Preparation and Application of Light-Colored Lignin Nanoparticles for Broad-Spectrum Sunscreens

Sang Cheon Lee ^1^, Eunjin Yoo ^2^, Sang Hyun Lee ^2^ and Keehoon Won ^1,*^

The active and inactive ingredients of the NIVEA refreshingly soft moisturizing cream:

Water, glycerin, myristyl alcohol, mineral oil, butylene glycol, alcohol denat., stearic acid, myristyl myristate, microcystaline wax, glyceryl stearate, hydrogenated coco-glycerides, simmondsia chinensis (jojoba) seed oil, tocopheryl acetate, lanolin alcohol, polyglyceryl-2 caprate, dimethicone, carbomer, phenoxyethanol, linalool, citronellol, alpha-isomethyl ionone, butylphenyl methylpropional, limonene, benzyl alcohol, benzyl salicylate, fragrance.

The active and inactive ingredients of the BIOTHERM lait solaire hydratant SPF 15:

Aqua/water, glycerin, alcohol denat., octocrylene, ethylhexyl salicylate, butyl methoxydibenzoylmethane, ethylhexyl triazone, diisopropyl sebacate, dimethicone, zea mays starch/corn starch, propylene glycol, triethanolamine, potassium cetyl phosphate, parfum/fragrance, synthetic wax, stearic acid, palmitic acid, PEG-100 stearate, glyceryl stearate, drometrizole trisiloxane, phenoxyethanol, terephthalylidene dicamphor sulfonic acid, caprylyl glycol, acrylates/C10-30 alkyl acrylate crosspolymer, tocopherol, xanthan gum, disodium EDTA, linalool, vitreoscilla ferment, myristic acid, geraniol, citral.

The active and inactive ingredients of the ATTITUDE 100% Mineral sunscreen SPF 15:

Zinc oxide, aqua, caprylic/capric triglyceride, cetearyl alcohol, cetearyl glucoside, glyceryl isostearate, glyceryl stearate, C10-18 triglycerides, glycerin, polyhydroxystearic acid, ethylhexyglycerin, capryl glycol, xanthan gum, sodium gluconate.
